# The Scorpion Toxin Analogue BmKTX-D33H as a Potential Kv1.3 Channel-Selective Immunomodulator for Autoimmune Diseases

**DOI:** 10.3390/toxins8040115

**Published:** 2016-04-19

**Authors:** Fang Ye, Youtian Hu, Weiwei Yu, Zili Xie, Jun Hu, Zhijian Cao, Wenxin Li, Yingliang Wu

**Affiliations:** State Key Laboratory of Virology, College of Life Sciences, Wuhan University, Wuhan 430072, China; fang.ye@whu.edu.cn (F.Y.); ythu@sjtu.edu.cn (Y.H.); Xrz-287@whu.edu.cn (W.Y.); zlxie@whu.edu.cn (Z.X.); Herohugo555@whu.edu.cn (J.H.); zjcao@whu.edu.cn (Z.C.)

**Keywords:** Kv1.3 channel, BmKTX, scorpion toxin, BmKTX-D33H, ion channel selectivity, autoimmune diseases

## Abstract

The Kv1.3 channel-acting scorpion toxins usually adopt the conserved anti-parallel β-sheet domain as the binding interface, but it remains challenging to discover some highly selective Kv1.3 channel-acting toxins. In this work, we investigated the pharmacological profile of the Kv1.3 channel-acting BmKTX-D33H, a structural analogue of the BmKTX scorpion toxin. Interestingly, BmKTX-D33H, with its conserved anti-parallel β-sheet domain as a Kv1.3 channel-interacting interface, exhibited more than 1000-fold selectivity towards the Kv1.3 channel as compared to other K^+^ channels (including Kv1.1, Kv1.2, Kv1.7, Kv11.1, KCa2.2, KCa2.3, and KCa3.1). As expected, BmKTX-D33H was found to inhibit the cytokine production and proliferation of both Jurkat cells and human T cells* in vitro*. It also significantly improved the delayed-type hypersensitivity (DTH) responses, an autoreactive T cell-mediated inflammation in rats. Amino acid sequence alignment and structural analysis strongly suggest that the “evolutionary” Gly11 residue of BmKTX-D33H interacts with the turret domain of Kv1 channels; it appears to be a pivotal amino acid residue with regard to the selectivity of BmKTX-D33H towards the Kv1.3 channel (in comparison with the highly homologous scorpion toxins). Together, our data indicate that BmKTX-D33H is a Kv1.3 channel–specific blocker. Finally, the remarkable selectivity of BmKTX-D33H highlights the great potential of evolutionary-guided peptide drug design in future studies.

## 1. Introduction

The voltage-gated Kv1.3 potassium channel is a well-recognized functional marker and attractive pharmacological target for treating autoimmune diseases [1,2,3,4,5]. The therapeutic efficacy of Kv1.3 channel blockers has been evidenced by* in vitro* assays of suppressing cytokine secretion and the proliferation of T cells, and by* in vivo* experiments on diverse animal models of autoimmune diseases (rheumatoid arthritis, multiple sclerosis (MS), type-1 diabetes mellitus (T1DM),* etc.*) [6,7,8,9,10]. Therefore, potent and selective Kv1.3 blockers have great pharmaceutical value in the treatment of autoimmune diseases.

**Figure 1:**
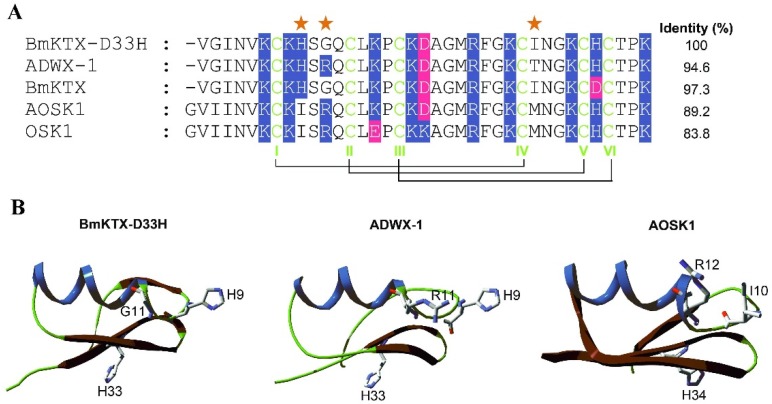
Alignment of amino acid sequences of toxins belonging to the α-KTx3 family. (**A**) The conserved half-cystine residues that form disulfide bonds are shown in green; basic residues are shown in blue shadow; acidic residues are shown in pink shadow. The three distinct amino acid residues (at positions 9, 11 and 28) between BmKTX-D33H and other relevant toxins are highlighted as “red stars”; (**B**) The locations of the main different amino acid residues between BmKTX-D33H and its structurally related toxins. The BmKTX-D33H structure is modeled using the BmKTX structure as its molecular template (PDB code: 1BKT); ADWX-1 structure (PDB code: 2K4U); AOSK1 structure (PDB code: 2CK4).

The venomous scorpions, which have lived on earth for over 400 million years [11], rely on their venoms for their efficient defense and capture strategy [12]. During their molecular evolution, scorpions produced hundreds of peptide-based toxins in their venoms, typically targeting ion channels with a high degree of molecular diversity [13,14]. Accordingly, scorpion venoms have been considered as invaluable resources of ion channel inhibitors. The genomic, transcriptomic and proteomic works have highlighted the great variety of amino acid sequences for scorpion toxins acting on K^+^ channels, although most of them share common three-dimensional structures [15,16,17,18,19,20,21]. In past years, a number of pharmacological experiments, in combination with mutagenesis and computational simulation techniques, have evidenced that most K^+^ channel–acting scorpion toxins are adopting the conserved anti-parallel β-sheet domains as their binding interfaces to interact with the Kv1.3 channel, including ChTX, OSK1, AgTX2, and ADWX-1 scorpion toxins [22,23,24,25,26,27,28,29,30,31]. Based on the interactions between scorpion toxins and potassium channels, a great effort has been made to find scorpion toxin peptides selectively acting on the Kv1.3 channel using molecular screening and design strategies. Among these toxin peptides, an analogue of scorpion toxin HsTX1 stabilized by four disulfide bridges was recently designed and found to be a selective blocker of the Kv1.3 channel [32]. Our group designed BmKTX-G11R/I28T/D33H (ADWX-1) and BmKTX-D33H toxin analogues starting from wild-type BmKTX scorpion toxin as the template [26,29]. Interestingly, ADWX-1 and BmKTX-D33H, which possess only two different amino acid residues, were both potent Kv1.3 blockers, with IC_50_ values of 1.9 and 15.4 pM, respectively [26,29]. Similar to the pharmacological profiles of the homologous toxin OSK1 and its analogue AOSK1 that simultaneously inhibit the Kv1.3 channel and other K^+^ channel subtypes, depending on the peptide concentration (Figure 1) [33], ADWX-1 also blocked the Kv1.1 channel with an IC_50_ value of 0.6 nM. Due to the similar binding modes of ADWX-1 and BmKTX-D33H which rely on their conserved anti-parallel β-sheet domains to interact with the Kv1.3 channel [26,29], whether or not potent BmKTX-D33H is a selective blocker of the Kv1.3 channel remained to be determined.

In this study, we investigated in detail the pharmacological profile of BmKTX-D33H. The electrophysiological data revealed that BmKTX-D33H actually exhibits a high selectivity for the Kv1.3 channel among a large series of potassium channel subtypes, including Kv1.1, Kv1.2, Kv1.7, Kv11.1, KCa2.2, KCa2.3, KCa3.1 channels. In addition to the desired selectivity, BmKTX-D33H acted as an effective immunosuppressant to modulate the cytokine secretion and proliferation of both Jurkat cells and human CD3^+^ T-cells. *In vivo*, it significantly ameliorated delayed-type hypersensitivity (DTH) responses in Lewis rats. In comparison with the highly homologous ADWX-1 and other scorpion toxins, the “unique” selectivity of BmKTX-D33H towards the Kv1.3 channel implies a functional evolution of the different amino acid residues among the structurally related scorpion toxins. This aspect might be key to the discovery of novel peptide drugs that selectively target the Kv1.3 channel.

## 2. Results

### 2.1. BmKTX-D33H is a Highly Selective Blocker of the Kv1.3 Channel

As reported previously by our group, scorpion toxin–derived BmKTX-D33H showed an increased potency (as compared to wild-type BmKTX) on the Kv1.3 channel, with an IC_50_ value of 15.4 pM [29]. It is also less potent than the structurally related ADWX-1, which shows an IC_50_ value of 1.9 pM on the same ion channel (Figure 1) [26]. Because ADWX-1 is a potent blocker of the Kv1.1 channel (IC_50_ value of 0.6 nM) [26], we further investigated the pharmacological profile of BmKTX-D33H.

**Figure 2:**
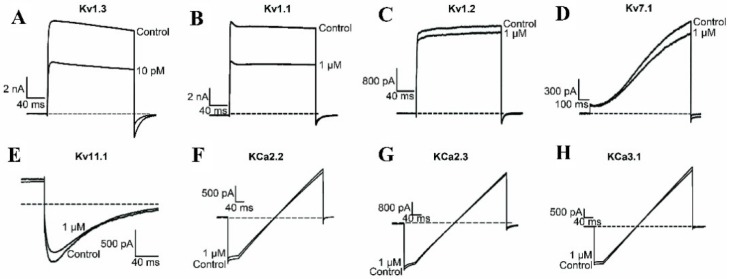
Pharmacological activities of BmKTX-D33H on different potassium channel subtypes. (**A**) Current traces in the absence (control) or presence of 10 pM BmKTX-D33H on the Kv1.3 channels; (**B**–**H**) Current traces in the absence (control) or presence of 1 μM BmKTX-D33H on the Kv1.1, Kv1.2, Kv7.1, Kv11.1, KCa2.2, KCa2.3, and KCa3.1 potassium channel subtypes, respectively. Data represent the means ± S.E. of at least three experiments.

The electrophysiological experiments indicate that BmKTX-D33H inhibits 56.0% ± 2.0% of Kv1.3 channel currents at a peptide concentration of 10 pM, whereas it only inhibits about 46.6% ± 2.0% of Kv1.1 channel currents at a concentration of 1 μM (Figure 2A,B). Interestingly, other potassium channel subtypes we examined, including the Kv1.2, Kv1.7, Kv11.1, KCa2.2, KCa2.3 and KCa3.1 channels, were much less sensitive to BmKTX-D33H action at its micromolar concentration (Figure 2C–H). In comparison with ADWX-1 toxin, the greater selectivity of BmKTX-D33H indicates it would be among the best candidate immunosuppressant drugs targeting the Kv1.3 channel.

### 2.2. Immunosuppressive Effect of BmKTX-D33H in Jurkat Cells

**Figure 3:**
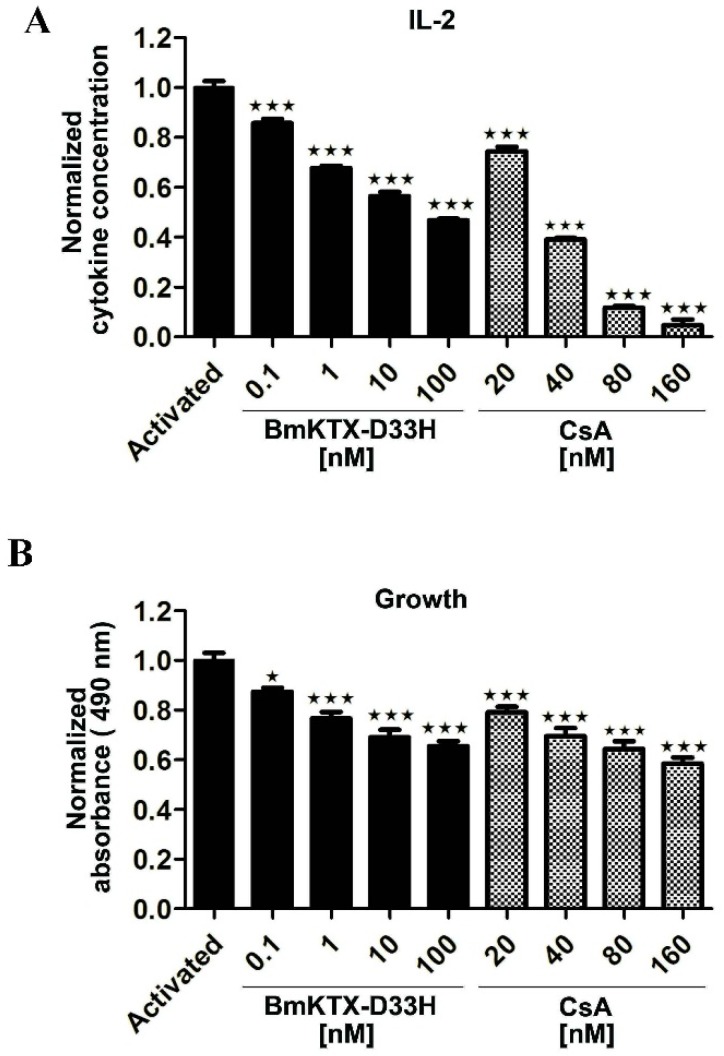
*In vitro* effect of BmKTX-D33H on Jurkat cell functions. Jurkat cells were stimulated with 100 ng/mL PMA and 1 mM Ionmycin. (**A**) Inhibition of IL-2 production in the absence or presence of BmKTX-D33H or CsA, respectively; (**B**) Proliferation of activated Jurkat cells was suppressed in the presence of BmKTX-D33H or CsA, respectively. *** *p* < 0.001, ** *p* < 0.01, * *p* < 0.05 *vs.* Control.

Potent and selective blockers of the Kv1.3 channel could efficiently inhibit both the cytokine secretion and proliferation of human T cells. Based on the “unique” pharmacological profile of BmKTX-D33H, we first employed Jurkat cells to confirm BmKTX-D33H potential to induce immunosuppression. As expected, our data demonstrate that BmKTX-D33H acts as an efficient immunosuppressant with regard to the activated Jurkat cells. As shown in Figure 3A and Table 1, IL-2 expression of Jurkat cells stimulated by PMA + ION was significantly reduced in the presence of BmKTX-D33H (IC_50_ value of 23.9 ± 20.1 nM). We also examined the effect of BmKTX-D33H on the proliferation of Jurkat cells at 48 h post-stimulation. It appears that the growth of Jurkat cells was also significantly inhibited in the presence of BmKTX-D33H (Figure 3B). For the sake of comparison, cyclosporin A (CsA, an immunosuppressant drug) was also found to inhibit both IL-2 secretion (IC_50_ value of 32.5 ± 0.4 nM) and proliferation of stimulated Jurkat cells (Figure 3 and Table 1). These results demonstrate the immunosuppressive action of BmKTX-D33H in Jurkat cells.

### 2.3. BmKTX-D33H Efficiently Inhibits Kv1.3 Channel Currents, Cytokine Secretion and Proliferation of Human T Cells

**Figure 4:**
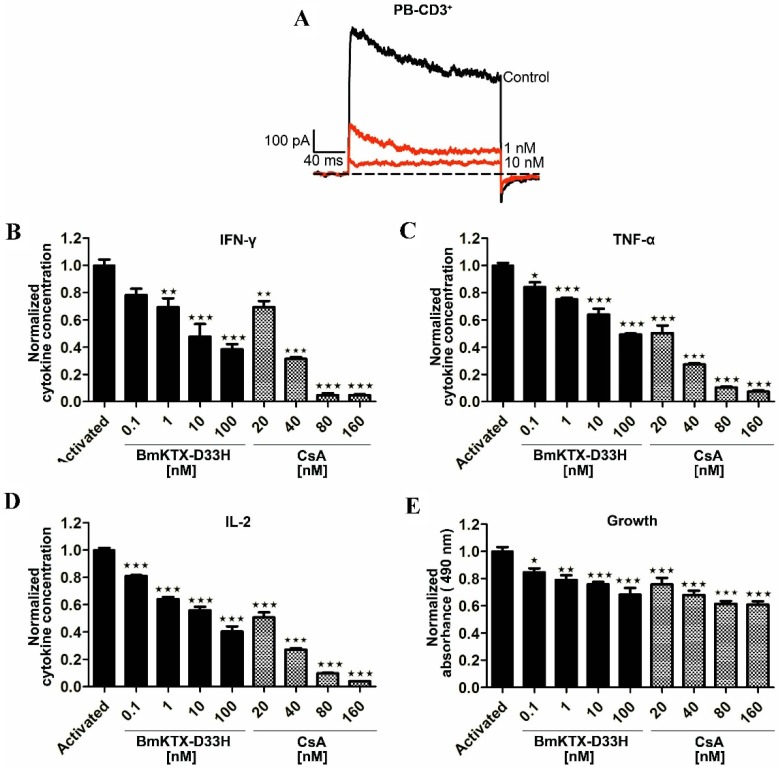
Pharmacological activities of BmKTX-D33H on Kv1.3 channels and immunomodulation in the primary human CD3^+^ T cells. (**A**) 63.3% ± 2.9% and 90.7% ± 2.1% of elicited Kv1.3 currents were inhibited by 1 and 10 nM BmKTX-D33H, respectively. Data represent the means ± S.E. of at least three experiments; (**B**–**E**) Suppression of cytokine production and* in vitro* proliferation of human CD3^+^ T cells induced by anti-CD3/CD28 beads in the presence of BmKTX-D33H or CsA, respectively. *** *p* < 0.001, ** *p* < 0.01, * *p* < 0.05.

To further evaluate the effect of BmKTX-D33H on human primary cells, we investigate the inhibitory potential of BmKTX-D33H on the Kv1.3 channels on the freshly isolated CD3^+^ T cells from healthy volunteers. As shown in Figure 4A, 1 and 10 nM BmKTX-D33H inhibited 63.3% ± 2.9% and 90.7% ± 2.1% of Kv1.3 currents, respectively. Based on such potent inhibition of the Kv1.3 channel currents by BmKTX-D33H, the immunosuppressive effects of BmKTX-D33H were further assessed through measuring the cytokine secretion of CD3^+^ T cells in the absence or presence of peptides. Pre-incubation of human T cells with BmKTX-D33H for 1 h before activation resulted in the robust dose-dependent reduction of IFN-γ (IC_50_ = 7.9 ± 7.8 nM), TNF-α (IC_50_ = 58.5 ± 48.6 nM) and IL-2 (IC_50_ = 11.2 ± 12.7 nM) secretions (Figure 4B–D and Table 1). Furthermore, BmKTX-D33H also suppressed the growth of CD3^+^ cells (Figure 4E). Also, CsA (immunosuppressant drug) was applied to human T cells. As shown in Figure 4 and Table 1, CsA suppressed the production of IFN-γ (IC_50_ = 28.3 ± 1.2 nM), TNF-α (IC_50_ = 20.0 ± 1.1 nM) and IL-2 (IC_50_ = 20.4 ± 0.4 nM), as well as the proliferation of human T cells. These findings support the immunosuppressive potential of BmKTX-D33H in human T cells.

### 2.4. BmKTX-D33H Inhibits DTH Reactions in Vivo

**Figure 5:**
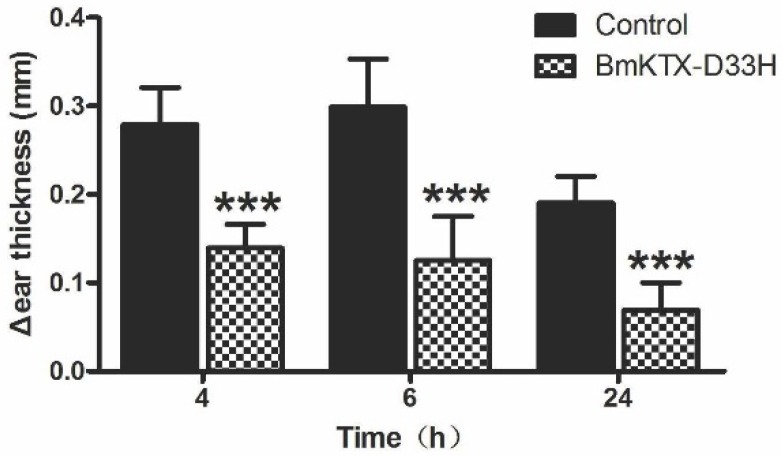
BmKTX-D33H exhibits immunosuppressive effects on delayed-type hypersensitivity in rats. Data are shown as differences in ear thickness between the ovalbumin-challenged ear and the saline-treated ear (mean ± S.E.). Measurement was performed at 4, 6, and 24 h after the challenge. The control group received a single subcutaneous injection of 0.5 mL vehicle (saline solution) in the scruff of the neck at the time of the second challenge. The drug treatment group received 100 μg/kg BmKTX-D33H (in 0.5 mL vehicle) administrated the same way. *** *p* < 0.001, ** *p* < 0.01, * *p* < 0.05.

Next, we evaluated the immunosuppressive potential of BmKTX-D33H* in vivo* by determining its capacity to inhibit the delayed-type hypersensitivity (DTH) reaction in Lewis rats. In our experiments, active DTH was evoked with ovalbumin immunization and subsequent ovalbumin challenge (seven days later), which resulted in swelling of the challenged ear. The DTH reaction was evaluated by measuring the ear swelling (change in ear thickness) at 4, 6, and 24 h after challenge. Figure 5 shows that the changes of ear thickness were significantly reduced in BmKTX-D33H-treated DTH rats, a distinct indication that the DTH response was ameliorated. These results demonstrate that BmKTX-D33H might be an efficient immunsuppressant* in vivo*.

## 3. Discussion

Because the Kv1.3 channel appears to be an important drug target in the management of various autoimmune diseases, the discovery of both potent and selective compounds (toxin and/or toxin-derived peptides) has been an active field in recent years [26,28,29,30,31,32,33]. Scorpion toxins are a well-known resource of Kv1.3 channel blockers, with IC_50_ values ranging from pM to μM concentrations. Interestingly, many so-called “classical” Kv1.3 channel–acting toxins are expected to adopt similar binding modes, in which the toxin anti-parallel β-sheets behave as binding interfaces [26,27,29,33,34,35,36]. However, it remains a challenge to find both a highly selective and potent scorpion toxin blocker of the Kv1.3 channel.

Here, we have investigated the selectivity and immunosuppressive potential of BmKTX-D33H, a very potent scorpion toxin–derived Kv1.3 channel blocker which adopts a conserved molecular interface to recognize the Kv1.3 channel [29]. Strikingly, BmKTX-D33H exhibits over 1000-fold selectivity for Kv1.3 channels (over many other potassium channel subtypes, such as Kv1.1, Kv1.2, Kv7.1, Kv11.1, KCa2.2, KCa2.3 and KCa3.1, see Figure 2). The particular structural and functional properties of BmKTX-D33H (as a Kv1.3 channel–specific blocker) make it a valuable immunosuppressant to modulate the immune responses of Jurkat cells and human CD3^+^ T cells (including inhibition of cytokine secretion and cell proliferation). In the DTH model, it was also demonstrated that the inflammatory responses of DTH rats were actually ameliorated by the BmKTX-D33H treatment. These data clearly indicate that BmKTX-D33H is a promising candidate drug for targeting the Kv1.3 channel.

**Figure 6:**
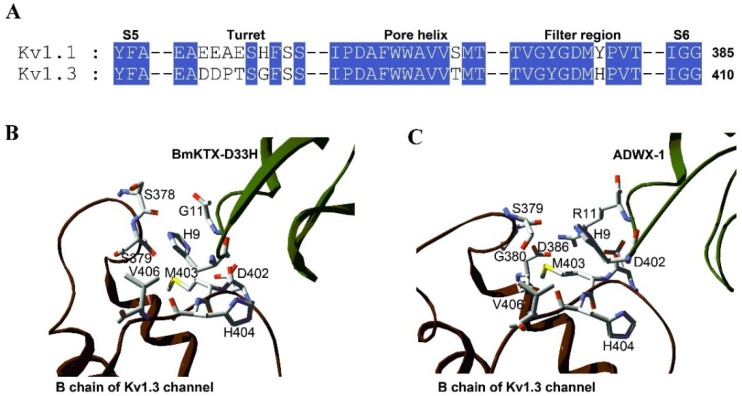
Comparison of the molecular interactions between BmKTX-D33H or ADWX-1 and the potassium channels. (**A**) The amino acid sequence alignment between Kv1.1 and Kv1.3 channel pore regions; (**B**) Interaction details of His9 and Gly11 residues in BmKTX-D33H with the Kv1.3 channel amino acid residues, within a contact distance of 5 Å. BmKTX-D33H-Kv1.3 complex from previous work [29]; (**C**) Interaction details of His9 and Arg11 residues in ADWX-1 with the Kv1.3 channel amino acid residues, within a contact distance of 5 Å. ADWX-1-Kv1.3 complex from previous work [26].

The most remarkable feature of BmKTX-D33H is its sought-after selectivity towards the Kv1.3 channel. Indeed, improving the selectivity of scorpion toxins (especially Kv1.1* versus* Kv1.3 channel subtypes) still remains a serious challenge [36]. Examples are provided with the Kv1.3 channel–acting ADWX-1, OSK1 and AOSK1, which are inhibitors of several Kv1.x channel subtypes at high nanomolar toxin concentrations. The alignment of amino acid sequences between BmKTX-D33H and ADWX-1 indicates that there are only two different amino acid residues, at toxin positions 11 and 28 (Figure 1). For ADWX-1, mutagenesis experiments demonstrate that the potency of ADWX-1-R11A and ADWX-1-T28A analogues on blocking the Kv1.3 channel were about 178- and 31-fold lower than that of native ADWX-1, indicating the importance of these two amino acid residues in toxin binding [26]. For BmKTX-D33H, the different Ile28 also showed a significant role in toxin binding since its substitution by an alanine residue decreases toxin-blocking potency by *ca.* 47-fold [29]. There is another different residue,* i.e*., Ile10 (which is adjacent to Arg12), in AOSK1 and OSK1 scorpion peptides (Ile10 is replaced by the His9 residue in both BmKTX-D33H and ADWX-1) (see Figure 1). Structurally, His9 and Gly11 residues of BmKTX-D33H are likely interacting with Ser378 and Ser379 (in the turret domain) and Asp402, Met403, His404 and Val406 (in the filter region) of the Kv1.3 channel, within a contact distance of 5 Å (Figure 6A,B). Such channel turret and filter region domains (especially the turret domain) were shown to be responsible for (classical) toxin selectivity (Kv1.1* versus* Kv1.3 channel subtype) [36]. Distinct from the interaction contacts of His9 and Gly11 in BmKTX-D33H, the ion channel residues interacting with His9 and Arg11 residues of ADWX-1 are expected to be the Asp386 and Gly380 residues which are located in the Kv1.3 channel turret (especially electrostatic interactions between toxin Arg11 and ion channel Asp386) (Figure 6C). Although Ile28 of BmKTX-D33H and Thr28 of ADWX-1 are likely playing an important role in toxin potency, these two amino acid residues are adjacent to the channel pore–blocking Lys26 residue, and are located above the ion channel filter region; they are, therefore, not thought to be crucial in toxin selectivity (Kv1.1* versus* Kv1.3 channel) [36]. This is presumably also why AOSK1 and OSK1 are able to block different Kv1.x channels at high nanomolar concentrations. In view of the Arg11 residue of ADWX-1 (conserved amino acid residue among scorpion toxins) (Figure 1) and the Asp386 residue of the Kv1.3 channel (conserved amino acid residue among Kv channels) (Figure 6), such distinct interactions between toxins and ion channel turrets might likely explain the higher selectivity of BmKTX-D33H towards the Kv1.3 channel (over other Kv channels).

In conclusion, BmKTX-D33H, with its conserved anti-parallel β-sheet domain as a binding interface, exhibits remarkably high blocking potency and selectivity for the Kv1.3 channels. It efficiently inhibits cytokine production and proliferation of both Jurkat cells and human T cells. Moreover, it dramatically reduced the* in vivo* DTH responses mediated by T cells in rat models. The “unique” and appropriate pharmacological properties of BmKTX-D33H make it a promising candidate drug for the management of autoimmune disorders. Also, this study on the Kv1.3 channel–specific BmKTX-D33H highlights the great potential of the evolutionary-guided peptide drug design strategy in future research investigations.

## 4. Experimental Section

### 4.1. Peptide Expression and Purification

Kv1.3 channel mutants were generated using the QuickChange Site-Directed Mutagenesis Kit (Stratagene, Santa Clara, CA, USA) based on wild-type pGEX-6P-BmKTX plasmid. The sequences of plasmids were verified by DNA sequencing before expression. BmKTX-D33H peptide was obtained according to a procedure described previously. The expression vector was transformed into *E.* *coli* Rosetta (DE3) cells, which were cultured in LB medium with ampicillin (100 μg/mL) at a temperature of 37 °C. When the desired cell density reached to OD = 0.6 (measured at a wavelength of 600 nm), 1.0 mM isopropyl-β-thio-b-d-galactoside (IPTG) was added to induce the expression at a temperature of 28 °C. The cells were harvested 4 h post-induction and resuspended into chilled 50 mM Tris-HCl buffer, pH 8.0, 10 nM Na_2_EDTA. After the cells were cracked using ultrasonic bath, the supernatant from the lysate was loaded to a GST-binding column. Purified fusion peptide was then desalted using centrifuge filtration (Millipore, Billerica, MA, USA) and cleaved by enterokinase (Biowisdon, Yantai, China) at a temperature of 25 °C for 16 h. Protein samples were then separated by reversed-phase HPLC on a C18 column (10 × 250 mm, 5 μm) (Elite-HPLC, Dalian, Liaoning, China) using a linear gradient from 10% to 80% acetonitrile with 0.1% trifluoroacetic acid, with detection at a wavelength of 230 nm. The molecular mass of the purified BmKTX-D33H was obtained by MALDI-TOF-MS (Voyager-DESTR, Applied Biosystems, Waltham, MA, USA). Peptide purity was >95%.

### 4.2. Electrophysiological Studies

Whole cell patch-clamp recordings of Kv1.3 channel were obtained from human T cells. Electrophysiological experiments of other assayed potassium channels were performed on HEK293 cells transfected with cDNA encoding Kv1.1, Kv1.2, Kv1.3, Kv7.1, Kv11.1, KCa2.2, KCa2.3, and KCa3.1 channels using TurboFect Reagent (ThermoSci., Waltham, MA, USA). The cDNAs were subcloned into the *Xho* I/*Bam *HI sites of pIRES2-EGFP, a bicistronic expression vector (Clontech, Mountain View, CA, USA) coexpressed with enhanced GFP. The constructs were verified by DNA sequencing. Currents were recorded on eGFP-expression positive cells one to three days post-transfection. Electrophysiological experiments were performed using protocols according to previously published references. Cells were bathed with mammalian Ringer’s solution: 5 mM KCl, 140 mM NaCl, 2 mM CaCl_2_, 1 mM MgCl_2_, 10 nM HEPES, 10 mM d-glucose (adjusted to pH 7.4 with NaOH). A multichannel microperfusion system MPS-2 (INBIO Inc, Wuhan, China) was used to exchange the external recording bath solution. The recording pipette solution contained 140 mM KCl, 1 mM MgCl_2_, 1 mM EGTA, 1 mM Na_2_ATP, 5 mM HEPES (adjusted to pH 7.4 with NaOH). All ion channel currents were elicited by depolarizing voltage steps of 200 ms from the holding potential −80 mV to +50 mV. Membrane currents were measured with an EPC 10 patch clamp amplifier (HEKA Elektronik, Lambrecht/Pfalz, Germany) interfaced to a computer running acquisition and analysis software (Pulse, v8.80, HEKA Elektronik, Lambrecht/Pfalz, Germany). Analysis of the data was performed with IgoPro (WaveMetrics, Lake Oswego, OR, USA). The results are mainly shown as the mean ± S.E. of at least three experiments.

### 4.3. Cell Preparation and Culture Condition

Jurkat E6-1 cells were obtained from the American Tissue Culture Collection (ATCC, Manassas, VA, USA) and cultured in RPMI-1640 supplied with 10% fetal bovine serum (FBS).

Peripheral blood (PB) obtained from four healthy volunteers was approved by the Ethics Committee of the College of Life Sciences in Wuhan University (Permit number: ECCLS20120076, approved at 3 May 2012). Ficoll gradients were used to isolate mononuclear cells from PB. The CD3^+^ T cells were then purified using EasySep Isolation Kit (StemCell Technologies, Vancouver, BC, Canada), and cultured at density of 5 × 10^5^ cells per cm^2^ in RPMI-1640 medium supplemented with 2 mM glutamine, and 10% FBS. All the T-cells were cultured at a temperature of 37 °C in a humidified 95% air and 5% CO_2_ atmosphere. The Jurkat cells were stimulated with 100 ng/ml phorbol ester (PMA) (Sigma-Aldrich, Munich, Germany) and 1 mM Ionmycin (ION) (Sigma-Aldrich, Munich, Germany). The CD3^+^ T cells were stimulated with anti-CD3/CD28 antibodies beads (Life Technologies, Waltham, MA, USA). BmKTX-D33H (ranged from 0.1 to 100 nM) was added for 1 h prior to stimulation.

HEK293 cells were cultured in Dulbecco’s Modified Eagle’s medium with 10% heat-inactivated fetal bovine serum (Hyclone, Logan, UT, USA).

### 4.4. Measurement of Cytokine Production and Cell Proliferation

The Jurkat cells or peripheral blood CD3^+^ cells were seeded in round-bottom plates (Corning-costar) at 5 × 10^5^ cells per cm^2^. BmKTX-D33H at various concentrations (0.1–100 nM) was added 1 h prior to stimulation. The cells were cultured for 48 h, and cell proliferation was measured using CellTiter 96 AQueous One Solution Cell Proliferation Assay Kit (Promega, Madison, WI, USA) in accordance with the manufacturer’s guide. Absorbance was read at a wavelength of 490 nm.

The IL-2, IFN-γ, and TNF-α levels in supernatants of stimulated cells were measured with ELISA kit (Dakewei, Shenzhen, China) following the manufacturer’s protocol. Absorbance was measured with an automatic plate reader at a wavelength of 450 nm with reference at 630 nm. As a comparison, the cyclosporin A (CsA) immunosuppressant drug (Sigma-Aldrich, Munich, Germany) was applied to the cells at different drug concentrations (20, 40, 80, and 160 nM, respectively).

### 4.5. Delayed-Type Hypersensitivity in Rats

The animal trial was approved by the Animal Use and Care Committee of the Wuhan University. Female Lewis rats (9–10 weeks of age) were purchased from Charles River Laboratories and housed under pathogen-free conditions. Active DTH was induced as describe previously by immunizing rats against antigen of ovalbumin (Sigma-Aldrich, Munich, Germany) emulsified with complete Freund’s adjuvant (Sigma-Aldrich, Munich, Germany) [37]. The emulsion was injected subcutaneously into Lewis rats in order to prime the immune system and develop memory T cells. Seven days later, the rats were challenged again with ovalbumin (20 μg in 20 μL of saline solution) in the pinna of one ear (challenged ear). The other side of ear received saline solution as challenge control. Rats in control group received a single subcutaneous injection of 0.5 mL vehicle (saline solution) in the scruff of the neck, at the time of second-challenge. Whereas, rats in drug-treatment group received 100 μg/kg BmKTX-D33H (in 0.5 mL vehicle) administrated using the same method. The change for thickness of ear (at 4, 6, and 24 h post-challenge) was measured as an indication of the inflammatory reaction and DTH severity.

### 4.6. Statistical Analysis

All data are expressed as mean ± S.E. Statistical analysis was carried out by using a one-way analysis of variance (ANOVA). A *p*-value less than 0.05 was considered significant.

## Figures and Tables

**Table 1:** Pharmacological activities of BmKTX-D33H and CsA on inhibiting cytokine secretion of Jurkat and CD3^+^ T cells.

Cell Types	Reagents (nM)	IC_50_ for Cytokines		
		IFN-γ	TNF-α	IL-2
Jurkat	BmKTX-D33H	NA	NA	23.9 ± 20.1
	CsA	NA	NA	32.5 ± 0.4
CD3^+^	BmKTX-D33H	7.9 ± 7.8	58.5 ± 48.6	11.2 ± 12.7
	CsA	28.3 ± 1.2	20.0 ± 1.1	20.4 ± 0.4

NA: not applicable.

## Abbreviations

DTH: Delayed type hypersensitivity
eGFP: Enhanced green fluorescent protein
h: Human
IPTG: Isopropyl thio-b-d-galactoside
ION: Ionmycin
MS: Multiple sclerosis
OSK1: *Orthochirus scrobiculosus* toxin 1
PB: Peripheral blood
PBS: Phosphate-buffered saline
BmKTX: Potassium channel blocking toxin from scorpion *Buthus martensii*
Asp^33^ to His, BmKTX-D33H: Potassium channel blocking toxin from scorpion *Buthus martensii *with a mutated position 33
PMA: Propylene glycol monomethyl ether acetate
ShK: *Stichodactyla helianthus* toxin
T1DM: Type-1 diabetes mellitus
CsA: Cyclosporin A

## References

[1] Wulff H., Calabresi P.A., Allie R., Yun S., Pennington M., Beeton C., Chandy K.G. (2003). The voltage-gated Kv1.3 K^+^ channel in effector memory T cells as new target for MS. J. Clin. Invest..

[2] Wulff H., Beeton C., Chandy K.G. (2003). Potassium channels as therapeutic targets for autoimmune disorders. Curr. Opin. Drug Discov. Devel..

[3] Chandy K.G., Wulff H., Beeton C., Pennington M., Gutman G.A., Cahalan M.D. (2004). K^+^ channels as targets for specific immunomodulation. Trends Pharmacol. Sci..

[4] Beeton C., Pennington M.W., Wulff H., Singh S., Nugent D., Crossley G., Khaytin I., Calabresi P.A., Chen C.Y., Gutman G.A. (2005). Targeting effector memory T cells with a selective peptide inhibitor of Kv1.3 channels for therapy of autoimmune diseases. Mol. Pharmacol..

[5] Beeton C., Wulff H., Standifer N.E., Azam P., Mullen K.M., Pennington M.W., Kolski-Andreaco A., Wei E., Grino A., Counts D.R. (2006). Kv1.3 channels are a therapeutic target for T cell-mediated autoimmune diseases. Proc. Natl. Acad. Sci. USA.

[6] Tudor J.E., Pallaghy P.K., Pennington M.W., Norton R.S. (1996). Solution structure of ShK toxin, a novel potassium channel inhibitor from a sea anemone. Nat. Struct. Biol..

[7] Kalman K., Pennington M.W., Lanigan M.D., Nguyen A., Rauer H., Mahnir V., Paschetto K., Kem W.R., Grissmer S., Gutman G.A. (1998). ShK-Dap22, a potent Kv1.3-specific immunosuppressive polypeptide. J. Biol. Chem..

[8] Schmitz A., Sankaranarayanan A., Azam P., Schmidt-Lassen K., Homerick D., Hansel W., Wulff H. (2005). Design of PAP-1, a selective small molecule Kv1.3 blocker, for the suppression of effector memory T cells in autoimmune diseases. Mol. Pharmacol..

[9] Li Z., Liu W.H., Han S., Peng B.W., Yin J., Wu Y.L., He X.H., Li W.X. (2012). Selective inhibition of CCR7^−^ effector memory T cell activation by a novel peptide targeting Kv1.3 channel in a rat experimental autoimmune encephalomyelitis model. J. Biol. Chem..

[10] Pennington M.W., Chang S.C., Chauhan S., Huq R., Tajhya R.B., Chhabra S., Norton R.S., Beeton C. (2015). Development of highly selective Kv1.3-blocking peptides based on the sea anemone peptide ShK. Mar. Drugs.

[11] Dunlop J.A., Kamenz C., Scholtz G. (2007). Reinterpreting the morphology of the Jurassic scorpion Liassoscorpionides. Arthropod Struct. Dev..

[12] Fry B.G., Roelants K., Champagne D.E., Scheib H., Tyndall J.D., King G.F., Nevalainen T.J., Norman J.A., Lewis R.J., Norton R.S. (2009). The toxicogenomic multiverse: Convergent recruitment of proteins into animal venoms. Annu. Rev. Genomics Hum. Genet..

[13] Mouhat S., Jouirou B., Mosbah A., De Waard M., Sabatier J.M. (2004). Diversity of folds in animal toxins acting on ion channels. Biochem. J..

[14] Mouhat S., Andreotti N., Jouirou B., Sabatier J.M. (2008). Animal toxins acting on voltage-gated potassium channels. Curr. Pharm. Des..

[15] Ma Y., Zhao Y., Zhao R., Zhang W., He Y., Wu Y., Cao Z., Guo L., Li W. (2010). Molecular diversity of toxic components from the scorpion *Heterometrus petersii* venom revealed by proteomic and transcriptome analysis. Proteomics.

[16] Ruiming Z., Yibao M., Yawen H., Zhiyong D., Yingliang W., Zhijian C., Wenxin L. (2010). Comparative venom gland transcriptome analysis of the scorpion *Lychas mucronatus* reveals intraspecific toxic gene diversity and new venomous components. BMC Genom..

[17] Ma Y., He Y., Zhao R., Wu Y., Li W., Cao Z. (2012). Extreme diversity of scorpion venom peptides and proteins revealed by transcriptomic analysis: Implication for proteome evolution of scorpion venom arsenal. J. Proteom..

[18] He Y., Zhao R., Di Z., Li Z., Xu X., Hong W., Wu Y., Zhao H., Li W., Cao Z. (2013). Molecular diversity of Chaerilidae venom peptides reveals the dynamic evolution of scorpion venom components from Buthidae to non-Buthidae. J. Proteom..

[19] Cao Z., Yu Y., Wu Y., Hao P., Di Z., He Y., Chen Z., Yang W., Shen Z., He X. (2013). The genome of *Mesobuthus martensii* reveals a unique adaptation model of arthropods. Nat. Commun..

[20] Cao Z., Di Z., Wu Y., Li W. (2014). Overview of scorpion species from China and their toxins. Toxins (Basel).

[21] Xu X., Duan Z., Di Z., He Y., Li J., Li Z., Xie C., Zeng X., Cao Z., Wu Y. (2014). Proteomic analysis of the venom from the scorpion *Mesobuthus martensii*. J. Proteom..

[22] Chen R., Robinson A., Gordon D., Chung S.H. (2011). Modeling the binding of three toxins to the voltage-gated potassium channel (Kv1.3). Biophys. J..

[23] Wu Y., Cao Z., Yi H., Jiang D., Mao X., Liu H., Li W. (2004). Simulation of the interaction between ScyTx and small conductance calcium-activated potassium channel by docking and MM-PBSA. Biophys. J..

[24] Yi H., Cao Z., Yin S., Dai C., Wu Y., Li W. (2007). Interaction simulation of hERG K^+^ channel with its specific BeKm-1 peptide: Insights into the selectivity of molecular recognition. J. Proteome Res..

[25] Yi H., Qiu S., Cao Z., Wu Y., Li W. (2008). Molecular basis of inhibitory peptide maurotoxin recognizing Kv1.2 channel explored by ZDOCK and molecular dynamic simulations. Proteins.

[26] Han S., Yi H., Yin S.J., Chen Z.Y., Liu H., Cao Z.J., Wu Y.L., Li W.X. (2008). Structural basis of a potent peptide inhibitor designed for Kv1.3 channel, a therapeutic target of autoimmune disease. J. Biol. Chem..

[27] Qiu S., Yi H., Liu H., Cao Z., Wu Y., Li W. (2009). Molecular Information of charybdotoxin blockade in the large conductance calcium-activated potassium channel. J. Chem. Inf. Model.

[28] Chen Z.Y., Hu Y.T., Yang W.S., He Y.W., Feng J., Wang B., Zhao R.M., Ding J.P., Cao Z.J., Li W.X. (2012). Hg1, novel peptide inhibitor specific for Kv1.3 channels from first scorpion Kunitz-type potassium channel toxin family. J. Biol. Chem..

[29] Chen Z., Hu Y., Hu J., Yang W., Sabatier J.M., De Waard M., Cao Z., Li W., Han S., Wu Y. (2014). Unusual binding mode of scorpion toxin BmKTX onto potassium channels relies on its distribution of acidic residues. Biochem. Biophys. Res. Commun..

[30] Chen Z., Hu Y., Hong J., Hu J., Yang W., Xiang F., Yang F., Xie Z., Cao Z., Li W. (2015). Toxin acidic residue evolutionary function-guided design of de novo peptide drugs for the immunotherapeutic target, the Kv1.3 channel. Sci. Rep..

[31] Mouhat S., Teodorescu G., Homerick D., Visan V., Wulff H., Wu Y., Grissmer S., Darbon H., de Waard M., Sabatier J.M. (2006). Pharmacological profiling of *Orthochirus scrobiculosus* toxin 1 analogs with a trimmed *N*-terminal domain. Mol. Pharmacol..

[32] Rashid M.H., Huq R., Tanner M.R., Chhabra S., Khoo K.K., Estrada R., Dhawan V., Chauhan S., Pennington M.W., Beeton C. (2014). A potent and Kv1.3-selective analogue of the scorpion toxin HsTX1 as a potential therapeutic for autoimmune diseases. Sci. Rep..

[33] Mouhat S., Visan V., Ananthakrishnan S., Wulff H., Andreotti N., Grissmer S., Darbon H., de Waard M., Sabatier J.M. (2005). K^+^ channel types targeted by synthetic OSK1, a toxin from *Orthochirus scrobiculosus* scorpion venom. Biochem. J..

[34] Eriksson M.A., Roux B. (2002). Modeling the structure of agitoxin in complex with the Shaker K^+^ channel: A computational approach based on experimental distance restraints extracted from thermodynamic mutant cycles. Biophys. J..

[35] Chen R., Chung S.H. (2012). Engineering a potent and specific blocker of voltage-gated potassium channel Kv1.3, a target for autoimmune diseases. Biochemistry.

[36] Yin S.J., Jiang L., Yi H., Han S., Yang D.W., Liu M.L., Liu H., Cao Z.J., Wu Y.L., Li W.X. (2008). Different residues in channel turret determining the selectivity of ADWX-1 inhibitor peptide between Kv1.1 and Kv1.3 channels. J. Proteome Res..

[37] Beeton C., Chandy K.G. (2007). Induction and monitoring of active delayed type hypersensitivity (DTH) in rats. J. Vis. Exp..

